# Global research hotspots and trends in the field of surgical treatment of congenital tracheal stenosis in infants and children over the past 40 years: A bibliometric and visualization study

**DOI:** 10.1097/MD.0000000000043143

**Published:** 2025-07-04

**Authors:** Jie Xu, Meng Chen, Xin Wang, Xiaobing Luo

**Affiliations:** a Department of Sports Medicine, Sichuan Provincial Orthopedics Hospital, Chengdu, China; b Department of Emergency Medicine, Nanchong Hospital of Traditional Chinese Medicine, Nanchong, China; c Health Science Center, Peking University, Peking, China.

**Keywords:** bibliometrics, CiteSpace, congenital, CTS, surgical treatment, tracheal stenosis, VOSviewer

## Abstract

Congenital tracheal stenosis (CTS) is a rare and highly fatal airway obstructive disorder. Recent years have seen the recognition of several forms of surgery as safe and effective means of improving the survival of children with CTS. It is important to complement the comprehensive and systematic pulse combing analysis of this research field. In order to give future researchers ideas and references, we examine the literature on the surgical treatment of CTS during the past 40 years and compile a summary of the cutting-edge trends and research hotspots in this area. A search was conducted through the Web of Science database’s core dataset for relevant publications pertaining to the surgical treatment of CTS from 1983 to 2024. CiteSpace 6.3, VOSviewer 1.6.18, Scimago Graphica 1.0.26, R-bibliometrix 4.6.1, and Pajek 5.16 tools were used for analysis and visualization. 327 publications total were found. With 82 papers, the US ranked first. Great Ormond Street Hospital for Children National Health Service Foundation Trust was ranked first for institutions with 24 papers. With 48 papers, the Journal of Pediatric Surgery had the top spot among journals, while Elliott MJ had the highest ranking among authors with 15. Study keywords were sliding tracheoplasty, congenital long tracheal stenosis, airway reconstruction, interventional procedures, airway stenting, intact tracheal rings, degradable airway stents, modified sliding tracheoplasty, trachelectomy, difficult intubation, extracorporeal membrane oxygenation, central airway stenosis, balloon-expandable metal stents, airway mucosa, Pierre Robin sequence, ventilation. The existing body of research indicates that there is still a great deal of untapped potential in the surgical treatment of CTS. The research hotspot is the clinical efficacy study of selecting the optimal timing and sequence of surgical procedures for the treatment of CTS combined with cardiovascular disease, the use of extracorporeal circulatory devices as an aid to reduce the surgical mortality rate, and the exploring preoperative predictive risk by reviewing patient cases to explore preoperative predictive risk factor studies. The reliability and feasibility of a new index to objectively assess tracheal flow function before and after surgery, new bioabsorbable scaffolds with longer degradation times and increased radial expansion, and new technologies such as visualization surgery.

## 1. Introduction

Congenital tracheal stenosis (CTS) is a structural obstructive disease. Depending on the severity of tracheal stenosis, most of the affected children have a variety of clinical symptoms such as wheezing, recurrent respiratory infections, respiratory distress, cyanosis, and even respiratory failure when they are <1 year old.^[1]^ If left untreated, the morbidity and mortality rate can be as high as 50% to 80%.^[2]^ In children with CTS, the narrowed segment of the trachea is often combined with a complete tracheal ring or tracheal chondromalacia. The complete tracheal ring is “O” shaped instead of the normal “C” shape. Tracheal chondromalacia means that the ratio of cartilaginous rings to membranous tissue is reduced to 2:1 to 3:1 (normal 4:1 to 5:1),^[3]^ or even the absence of cartilaginous rings to form a cartilaginous sleeve.^[4]^ There is still a lack of standardized criteria for the classification of CTS. Clinically, the length of tracheal stenosis is used as the basis for better guiding the surgical approach. Tracheal stenosis less than or >50% of the total length of the main trachea was defined as short or long stenosis, respectively.^[5,6]^ Simple CTS is rare in clinical practice, accounting for only 10% to 30% of all children with tracheal stenosis.^[7]^ In children with CTS, there is often a combination of congenital cardiovascular diseases. The most common of these are combined with a pulmonary artery sling to form a “ring sling complex.”^[8]^ Therefore, the severity of tracheal stenosis in children depends on the internal diameter of the main tracheal stenosis segment, the length of the tracheal stenosis, whether the bronchus is involved, the morphology of the trachea, the presence of a complete tracheal ring, and the presence of other cardiovascular diseases in combination. Surgery is currently the predominant treatment modality for CTS. Common surgical approaches include rib cartilage,^[9,10]^ pericardium,^[11,12]^ trachea,^[13]^ esophagus,^[14]^ periosteum^[15]^ Patch grafting, Resection of stenotic segment of trachea with end-to-end anastomosis,^[16]^ Sliding tracheoplasty,^[17]^ Anterior tracheal wall suspension plasty,^[18]^ Expandable metal airway stenting,^[19]^ Biodegradable material airway stent placement^[20]^ and stem cell tracheal replacement.^[21]^ The mainstay technique for short-segment CTS is stenotomy with end-to-end anastomosis, and for long-segment CTS is slide tracheoplasty (STP).^[17,22]^ The diversity of clinical manifestations and the complexity of tracheal morphology pose a serious challenge to the timely and effective treatment of CTS. In recent years, computational fluid dynamics (CFD) has been accepted by many scholars and clinicians as an advanced numerical simulation tool. Simulation of airflow movement in the airway by computer technology can obtain parameters that cannot be captured by existing clinical techniques, among which the airway pressure difference can objectively measure airway resistance and ventilation efficiency,^[23,24]^ and the energy flow response is positively correlated with the clinical respiratory status in the preoperative and postoperative period.^[25]^ In addition, tissue-engineered trachea is also a hot and difficult research topic in this field. Among them, the selection of seed cells and scaffold materials, as well as the selection of culture conditions for tracheal prostheses are key issues.^[21,26]^

Clinical research on CTS surgical treatment has made great strides over the past 4 decades, and there is a consensus on its efficacy, but additional investigation is necessary to enhance our knowledge of the pathogenesis of CTS. As new theories and technologies emerge, the treatment of CTS presents both fresh opportunities and challenges. While investigating research trends and hotspots in the surgical treatment of CTS is crucial, there is currently no bibliometric analysis available in this area. This study aims to provide new perspectives for a rapid and in-depth understanding of the field, providing scholars with helpful resources and assistance.

## 2. Methods

### 2.1. Data retrieval and search strategy

**Figure 1.**
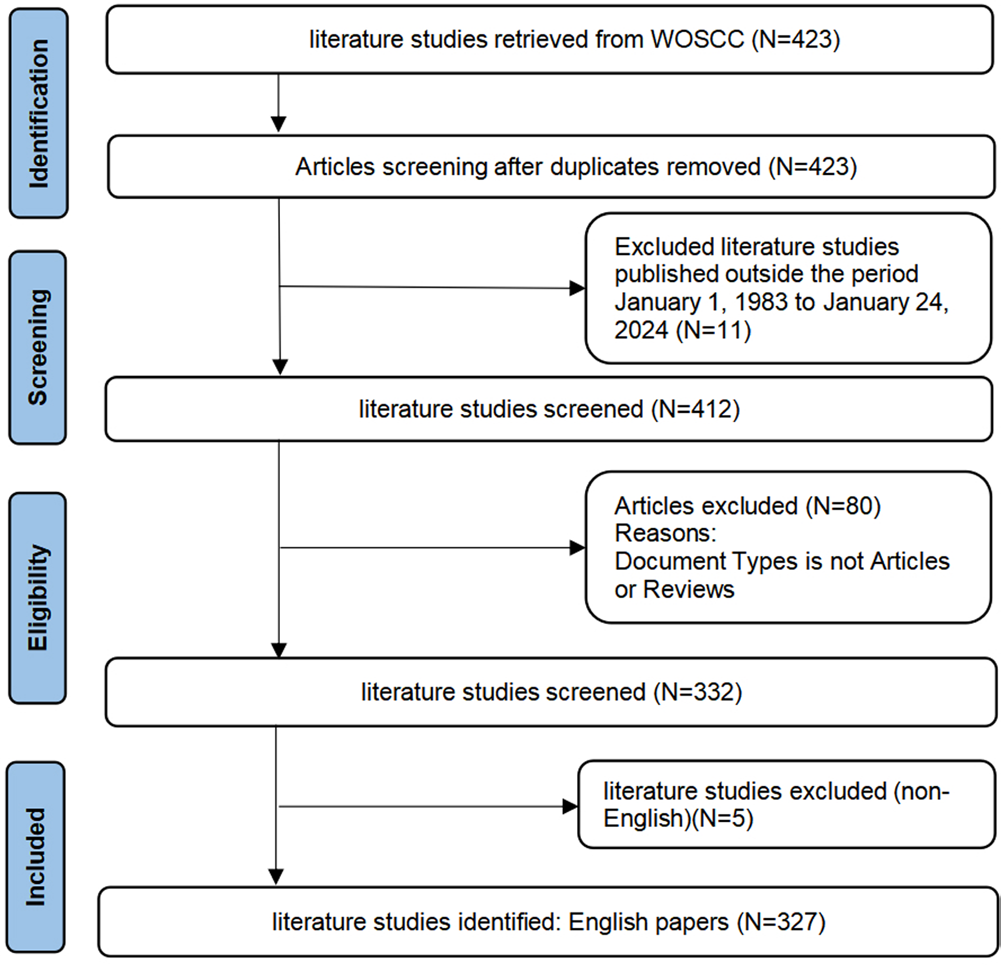
Workflow diagram.

One quantitative technique for assessing and analyzing scientific literature is bibliometric research. We created 1 file for every 500 records by exporting the Web of Science data in “plain text” format with “complete records and references.” After renaming the file to “download,” we placed it in CiteSpace software’s input folder and proceeded with the visualization study. Once node types like country (region), institution, author, cited author, journal, keyword, and cited literature are chosen for co-occurrence or cluster analysis, the visualization map can be created. Table S1, Supplemental Digital Content, https://links.lww.com/MD/P329, contains comprehensive details on the search strategy. The literature selection for this study adhered to these criteria: only Articles and Reviews were included; publication dates ranged from January 1, 1983, to January 24, 2024; literature had to be in English; there were no restrictions on species or organisms; duplicates were removed to maintain dataset uniqueness. No ethical approval was required, as the articles contained no personal patient information. Figure 1 displays the pertinent workflow diagram.

### 2.2. Literature screening

Two reviewers independently evaluated the literature. The initial screening involved evaluating titles and abstracts according to the inclusion criteria. A third assessor examined the entire work and made the final determination in the event of a disagreement.

### 2.3. Statistical analysis methods

Our bibliometric analysis study utilized several tools, including CiteSpace 6.3.R1, VOSviewer 1.6.18, R-Studio with R-bibliometrix 4.6.1, Pajek 5.16, and Scimago Graphica 1.0.26.

CiteSpace (Drexel University, Philadelphia), developed by Prof Chen C., is a software program that analyzes citation networks and evolutionary patterns in academic literature to identify research hotspots and emerging trends. Key research themes and highly referenced publications can be found with the aid of this tool. We employed CiteSpace for visual analysis, utilizing its features to examine journal citations, reference analysis, distribution by country and institution, as well as keyword and citation bursts. The detailed settings are as follows: the time range is set from 1983 to 2024. The filtering criterion is set to “Top N(50).” Choose “Pathfinder” as the method for trimming connections, clustering and labeling select All in One. All settings are provided in Table S2, Supplemental Digital Content, https://links.lww.com/MD/P330.

VOSviewer (Leiden University, Leiden, the Netherlands) is a visualization software developed by Prof Van Eck and Prof Waltman, which visualizes and analyzes co-occurrence network graphs, frequencies, and time-series information among the literature to discover research themes, evolutionary processes, and future trends, respectively.^[27]^ The detailed settings are as follows: normalization method selects “association strength,” random starts select 1, max. Iterations select 1000, merge small clusters select “yes,” and weights select “occurrences.” All settings are provided in Table S3, Supplemental Digital Content, https://links.lww.com/MD/P331.

R-bibliometrix, Pajek, and Scimago Graphica are powerful tools for multidimensional and multifaceted geographic visualization. We use them to highlight the collaboration networks between different countries or regions.

## 3. Results

### 3.1. Publication volume trends and characteristics

**Figure 2.**
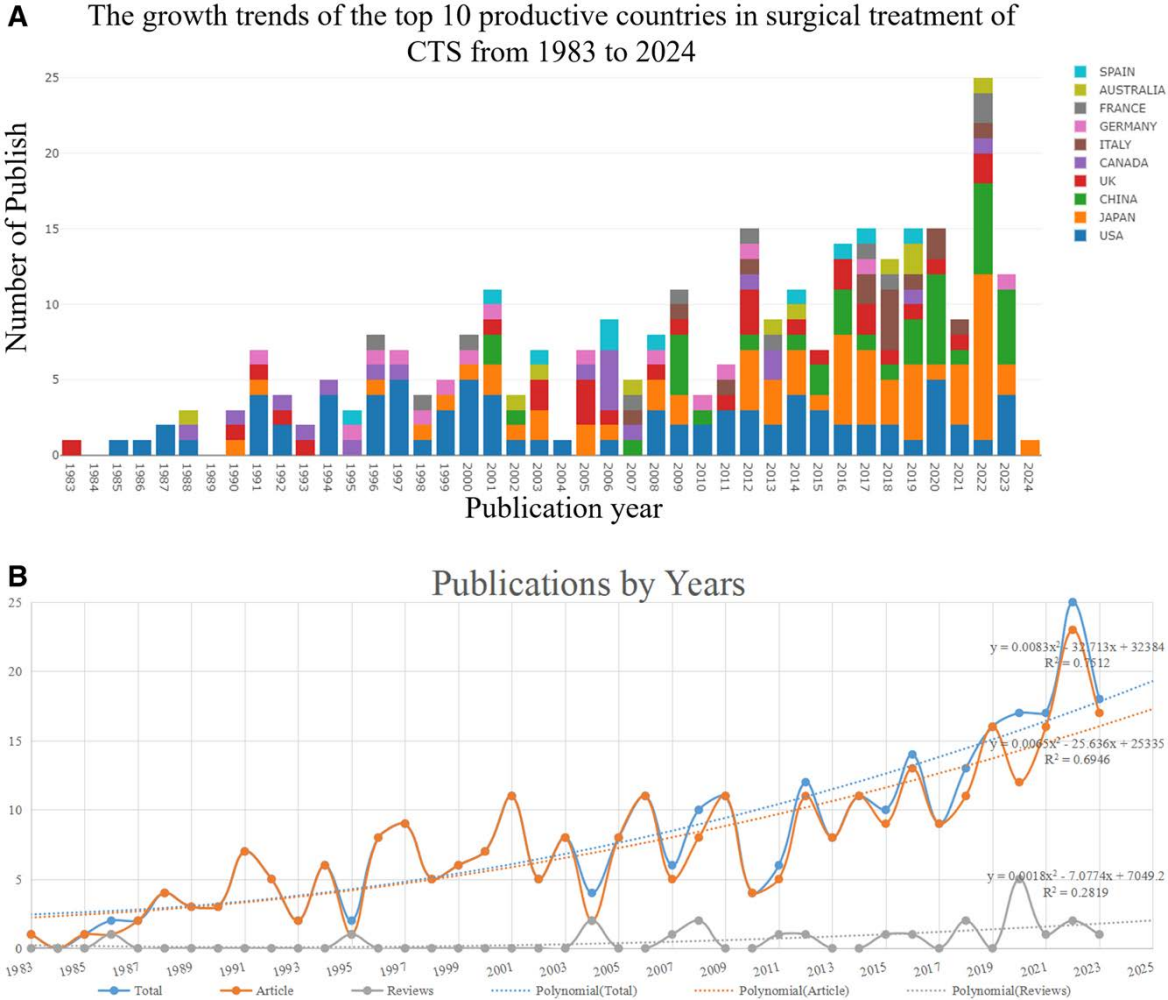
(A) Histogram of annual national publications. (B) Trends in publications and corresponding polynomial fit curves.

Using the Web of Science core database, 423 publications pertaining to surgical treatment of CTS field research were found, 11 kinds of literature whose publication time was not from January 1, 1983, to January 24, 2024, were excluded, then 80 literature whose literature type was not Article and Review were excluded, and finally, 5 non-English kinds of literature were excluded, and finally, 327 literatures were included. Literature, as shown in Flowchart 1. Among them, 305 original research pieces made up 93.27%, and 22 review papers made up 6.73%. The average number of citations per manuscript was 7.8, with a total citation frequency of 2550. Figure 2A presents the yearly publication volume from several countries. The number of research papers in the field remained relatively stable in general from 1983 to 2011, with the average number of publications per year remaining at 5.41. From 2012 to 2023 the number of publications rises to a new level, maintaining an average of 14.17 publications per year and peaking at 25 in 2022. Overall, the main countries conducting research in this area are the US, Japan, China, and the UK. The US and the UK were the first major nations to begin research in this field, and the US has the highest share of publications overall. Japan and China have steadily increased their share of publications annually, and the US, Japan, and China are consistently the top 3 nations in this field. A significant relationship was found through polynomial fitting analysis between the number of publications and the publication year, with R² values of 0.7512 for total papers, 0.6946 for articles, and 0.2819 for reviews. As seen in Figure 2B, we projected that the number of published papers would reach around 19 in 2025, comprising approximately 17 original articles and 2 reviews, based on the polynomial fitting analysis.

### 3.2. Countries or regions collaboration analysis

**Figure 3.**
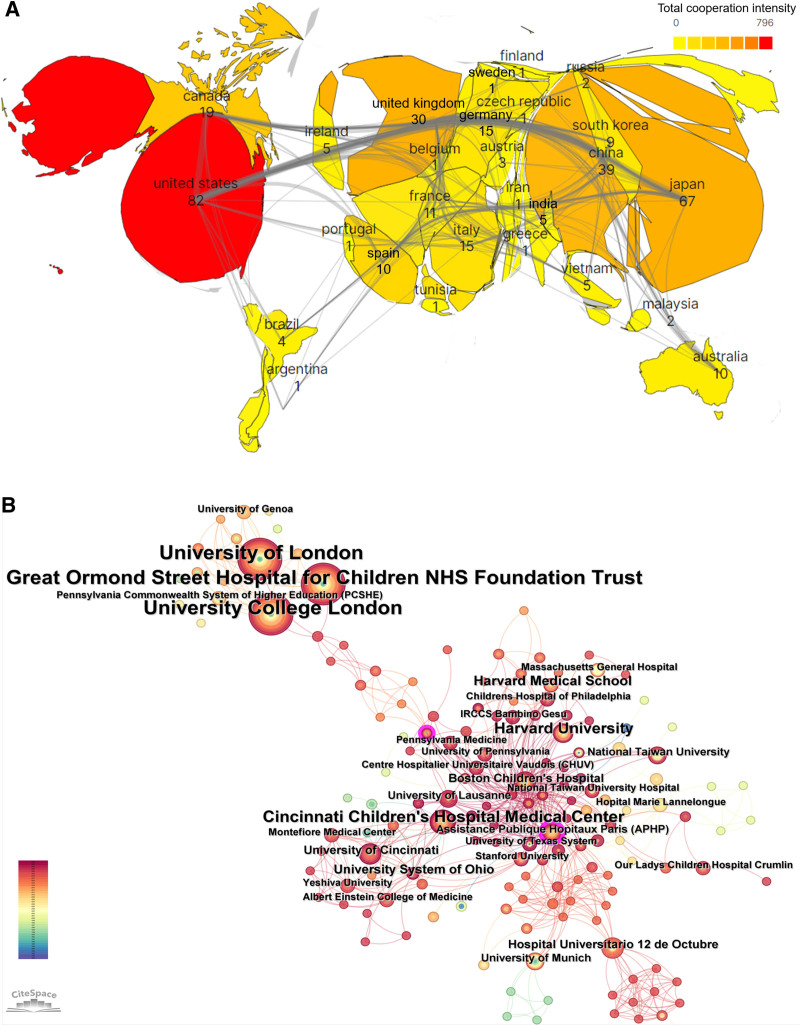
(A) Populated chart of the bibliometric analysis of country cooperation in the field of surgical treatment of CTS. (B) Map of the intensity of cooperation among agencies in the field of surgical treatment of CTS. CTS = congenital tracheal stenosis.

The resulting visualization map included 35 nations or regions, with 15 of them having published at least 5 articles. Table 1 presents data on the top 10 nations by total number of papers published. In Figure 3A, the size of each country’s area corresponds to the volume of articles published, with larger areas indicating higher publication volumes. Darker colors represent stronger cooperation relationships, while the thickness of the lines reflects the intensity of these links. The connecting lines illustrate the level of collaboration between countries. The nation with the most articles published was the US (82 articles or 25.08%), followed by Japan (67 articles or 20.49%) and China (39 articles or 11.93%). These 3 nations are the leading contributors to the subject; the total number of publications they have produced accounts for 57.49% of all publications. The US leads globally in total citation rate (TC), H-index, and total link strength (TLS), whereas Germany excels in average citations per publication (ACPP). TLS reflects the level of reciprocal cooperation, while the H-index is commonly used to assess academic impact.

**Table 1:** Top 10 high-impact countries in the field of surgical treatment of CTS.

Country	NP	TC	ACPP	TLS	H-index
USA	82 (25.08%)	1987	24.23	796	28
Japan	67 (20.49%)	740	11.04	432	14
China	39 (11.93%)	364	9.33	380	12
UK	30 (9.17%)	1104	36.80	416	16
Canada	19 (5.81%)	579	30.47	299	11
Italy	15 (4.59%)	210	14.00	152	7
Germany	15 (4.59%)	730	48.67	129	11
France	11 (3.36%)	151	13.73	106	7
Spain	10 (3.06%)	446	44.60	208	9
Australia	10 (3.06%)	93	9.30	68	5

ACPP = average citations per publication, CTS = congenital tracheal stenosis, NP = number of publications, TC = total citation, TLS = total link strength.

### 3.3. Research institution collaboration analysis

Among 333 institutions that published research on the surgical treatment of CTS, 24 had 5 or more publications. Table 2 provides data on the top 10 institutions, which represent 41.59% of the total publication output. Figure 3B depicts international cooperation among institutions, with circle sizes representing the number of publications by each nation. The connecting lines show the degree of cooperation, and the thickness of the lines shows how strongly the connections are made. It is worth noting that 3 institutions in the UK, University of London, Great Ormond Street Hospital for Children National Health Service (NHS) Foundation Trust, and University College London, together published 24 papers, which ranked top in the area for publications, making up 7.34% of all the papers published, followed by Kobe Childrens Hosp in Japan (17 papers, 5.20%) and Ann & Robert H. Lurie Children’s Hospital of Chicago in the US (15 papers, 4.59%). It is worth noting that University of London, Great Ormond Street Hospital for Children NHS Foundation Trust, and University College London in the UK collectively had the highest TC, ACPP, TLS, and H indices in terms of are the highest. Harvard University, Cincinnati Children’s Hospital Medical Center, and University of London have formed stronger research collaborations with several other institutions.

**Table 2:** Top 10 high-impact institutions in the field of surgical treatment of CTS.

Institution	NP	TC	ACPP	TLS	H-index	Location
Great Ormond Street Hospital for Children NHS Foundation Trust	24 (7.34%)	1015	42.31	573	15	UK
University of London	24 (7.34%)	1015	42.31	573	15	UK
University College London	24 (7.34%)	1015	42.31	573	15	UK
Kobe Childrens Hosp	17 (5.20%)	249	14.64	393	12	Japan
Ann & Robert H. Lurie Children’s Hospital of Chicago	15 (4.59%)	524	34.93	345	13	USA
Cincinnati Children’s Hospital Medical Center	14 (4.28%)	385	27.5	284	10	USA
Shanghai Jiao Tong University	13 (3.98%)	153	11.77	238	7	China
University of Toronto	13 (3.98%)	522	40.15	390	9	Canada
Hospital for Sick Children (SickKids)	13 (3.67%)	522	40.15	428	9	Canada
Harvard University	11 (3.36%)	399	36.27	363	5	USA

ACPP = average citations per publication, CTS = congenital tracheal stenosis, NHS = National Health Service, NP = number of publications, TC = total citation, TLS = total link strength.

### 3.4. High-impact author’s collaboration analysis

**Figure 4.**
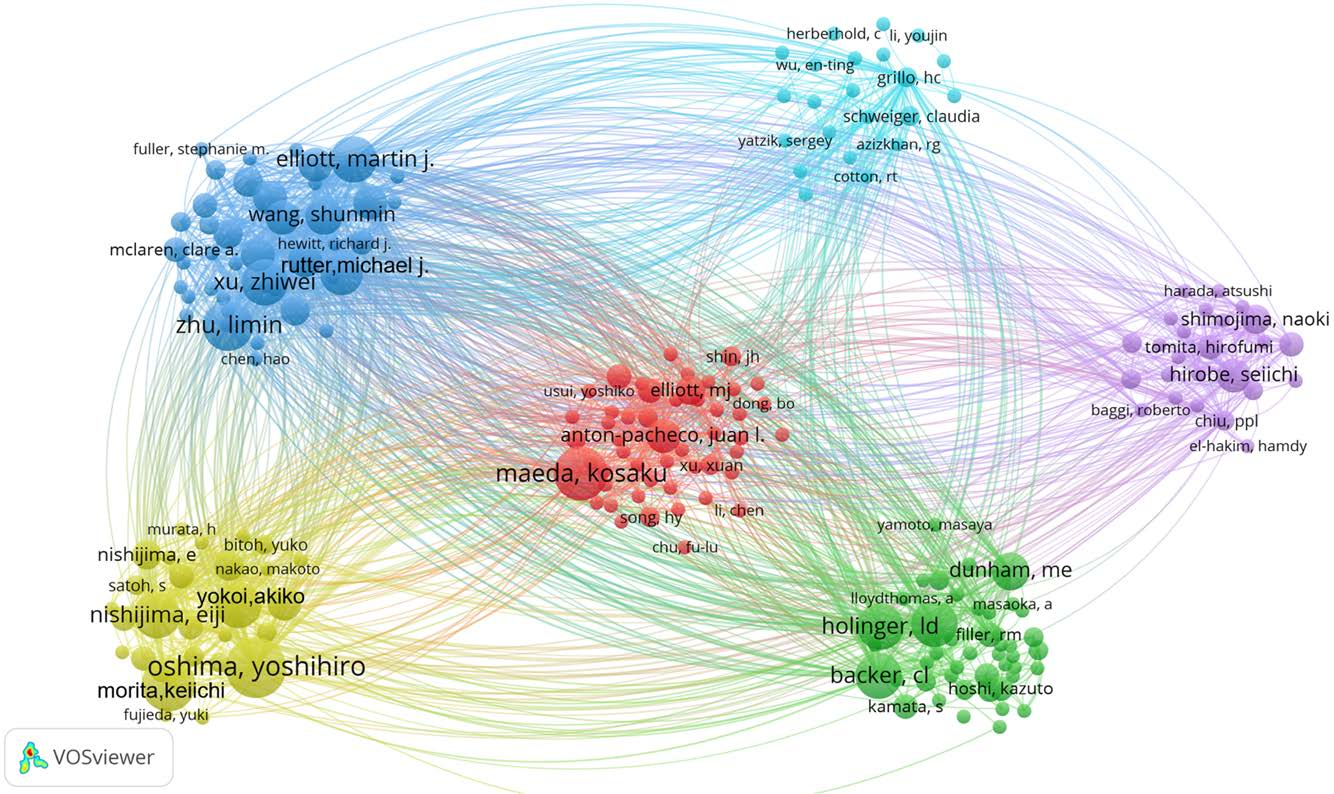
Author co-occurrence network diagram in the field of surgical treatment of CTS (http://tinyurl.com/yrhv5mgr). CTS = congenital tracheal stenosis.

Out of 1341 authors represented in the visualization map, 33 had published 5 or more articles. Table 3 presents the top 10 scholars ranked by their number of publications and citations. Figure 4 shows the extent of cooperation between author teams, with stronger connections observed among the high-producing authors. Three of the most prominent researchers in the field, i.e., Elliott MJ, Nishijima E, and Oshima Y, with 15 (4.59%), 14 (4.28%), and 14 (4.28%) publications respectively, are very active and influential authors. Elliott MJ of Great Ormond Street Hospital for Children NHS Foundation Trust, UK topped the TC, ACPP, H-index, and TLS. Grillo HC (228), Backer CL (160), and Antón-Pacheco JL (100) were the top 3 authors in the field in terms of co-citation frequency, based on the VOSviewer author co-citation analysis.

**Table 3:** Top 10 high-impact authors in the field of surgical treatment of CTS.

Author	NP	TC	ACPP	TLS	H-index	Country
Elliott MJ	15 (4.59%)	788	52.83	1217	12	UK
Nishijima E	14 (4.28%)	314	22.43	1106	11	Japan
Oshima Y	14 (4.28%)	231	16.5	1043	9	Japan
Maeda K	13 (3.98%)	119	9.18	564	7	Japan
Backer CL	12 (3.67%)	482	40.17	1039	11	USA
Holinger LD	11 (3.36%)	446	40.55	945	10	USA
Morita K	11 (3.36%)	44	4	628	3	Japan
Mavroudis C	10 (3.06%)	371	37.13	1033	8	Cyprus
Xu ZW	10 (3.06%)	194	19.43	702	6	China
Zhu LM	10 (3.06%)	70	7	782	4	China

ACPP = average citations per publication, CTS = congenital tracheal stenosis, NP = number of publications, TC = total citation, TLS = total link strength.

### 3.5. High-impact journals collaboration analysis

**Figure 5.**
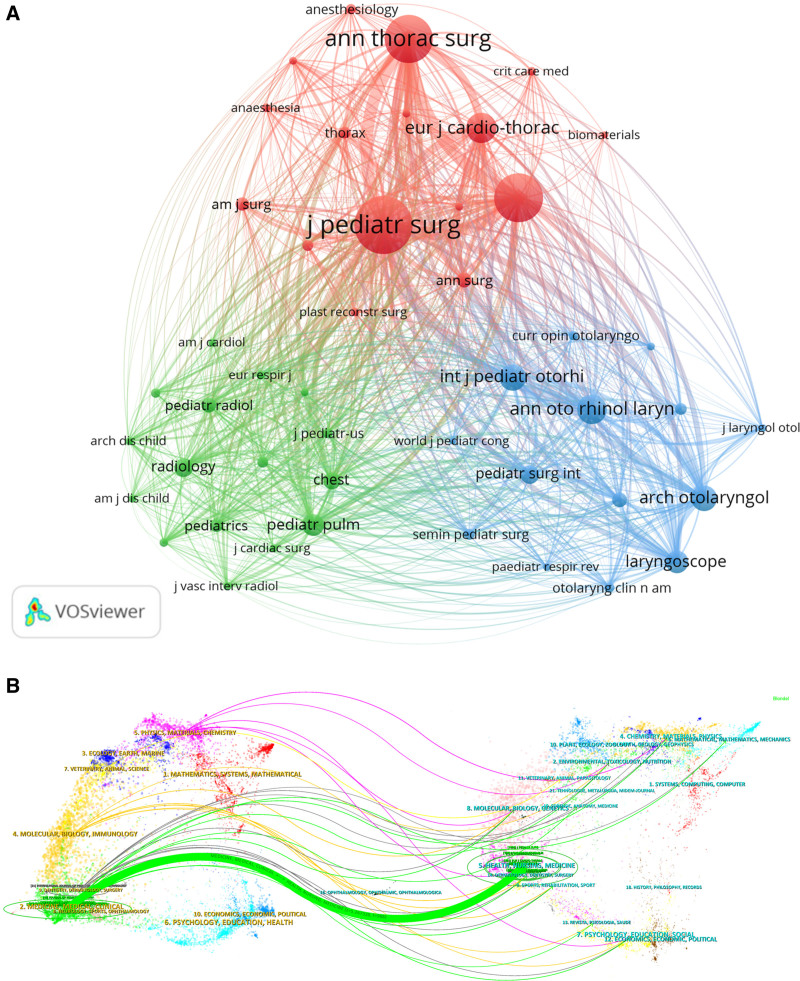
(A) Cluster visualization of journal co-citation analysis generated based on VOSviewer software. (http://tinyurl.com/272jue35). (B) Double plot overlay of citing and cited journals in the field of surgical treatment of CTS. Note: Cited journals on the left, cited journals on the right, and the connecting lines represent citations. CTS = congenital tracheal stenosis.

The 327 articles retrieved were from 116 journals, and according to the number of papers published, the top 3 journals were the Journal of Pediatric Surgery (48 articles, total citations 1306, mean citations 27.21), Pediatric Surgery International (25 articles, total citations 238, mean citations 9.52 citations) and Annals of Thoracic Surgery (22 articles, 655 total citations, 29.77 average citations). According to the H-index, the top 3 journals were the Journal of Pediatric Surgery (21), Annals of Thoracic Surgery (14), and European Journal of Cardio-Thoracic Surgery (12). Of these 10 journals, 70% are categorized as Q1 or Q2, suggesting a rather good caliber of research in the area. Since 90% of these 10 publications have their roots in Europe and the US, it is possible that they are essential to the advancement of the field’s studies. The top 3 co-cited journals for research on the surgical treatment of CTS, as identified through VOSviewer’s journal co-citation analysis, are the Journal of Pediatric Surgery (1082 citations), Journal of Thoracic and Cardiovascular Surgery (755 citations), and Annals of Thoracic Surgery (730 citations). Figure 5A and Table 4 display the network of connections between co-cited journals, comprising 46 journals with a citation frequency of 20 or more. A total of 3 clusters were generated in Figure 5A; the red cluster represents thoracic and cardio-thoracic surgery research journals with topics focused on thoracic and cardio-thoracic surgical procedures involving combined repair surgical strategies for complex cardiac malformations complicated by CTS. The green cluster represents pediatric and radiology research journals with topics focused on the diagnosis and preoperative evaluation of children with CTS. The blue cluster represents otolaryngology and airway reconstruction research journals, with topics focused on airway reconstruction and airway management. This highlights the significant impact of journal publishing in this field. The colored pathways in the double graph overlay illustrate citation linkages between the citing and cited journals. Studies published in HEALTH/NURSING/MEDICINE publications are typically cited by studies published in MEDICINE/MEDICAL/CLINICAL journals, as indicated by the colored routes. Figure 5B provides further details on the typical citing and referenced journals in each cluster. The Journal of Pediatric Surgery, the Journal of Thoracic and Cardiovascular Surgery, and the Annals of Thoracic Surgery, for instance, are the most illustrative publications in the HEALTH/NURSING/MEDICINE cluster.

**Table 4:** Top 10 high-volume journals in the field of surgical treatment of CTS.

Journal	NP	TC	ACPP	TLS	H-index	IF (2023)	JCR (2023)
Journal of Pediatric Surgery	48	1306	27.21	530	21	2.4	Q2
Pediatric Surgery International	25	238	9.52	229	9	1.8	Q3
Annals of Thoracic Surgery	22	655	29.77	330	14	4.6	Q1
International Journal of Pediatric Otorhinolaryngology	20	245	12.25	220	9	1.5	Q3
European Journal of Cardio-Thoracic Surgery	19	495	26.05	309	12	3.4	Q1
Journal of Thoracic and Cardiovascular Surgery	13	718	55.23	315	11	6	Q1
Pediatric Pulmonology	10	199	19.90	64	7	3.1	Q2
Frontiers in Pediatrics	7	61	8.71	78	3	2.6	Q2
Laryngoscope	7	29	4.14	37	5	2.6	Q2
Journal of Thoracic Disease	6	68	11.33	64	5	2.5	Q3

ACPP = average citations per publication, CTS = congenital tracheal stenosis, IF = impact factor, JCR = Journal Citation Reports, NP = number of publications, TC = total citation, TLS = total link strength .

## 4. Keyword analysis and visualization

**Figure 6.**
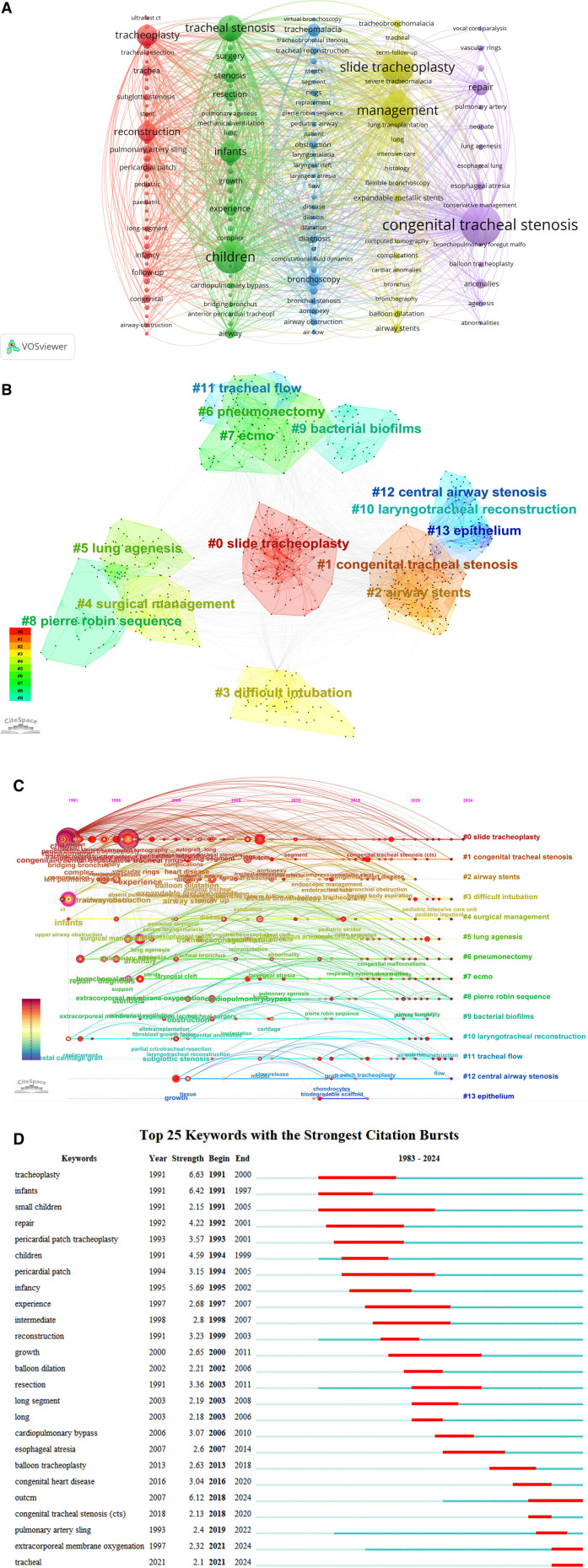
(A) Keyword view in the field of surgical treatment of CTS based on VOSviewer. (http://tinyurl.com/28cfxv3b). (B) CiteSpace-based keyword co-occurrence clustering in the field of surgical treatment of CTS. (C) Timeline view of keywords in the field of surgical treatment of CTS. (D) Keyword emergence map in the field of surgical treatment of CTS. CTS = congenital tracheal stenosis.

Keyword analysis is essential for exploring research trends and summarizing research hotspots. In Figure 6A, generated by VOSviewer, each vertical bar represents a cluster, each circle denotes a keyword, and the connecting lines illustrate relationships between keywords. Larger circles indicate higher term frequencies. Research hotspots are represented by the terms with high centrality and frequency listed in Table 5. Figure 6B shows the keyword co-occurrence clustering map for this field. Using the traditional log-likelihood ratio technique, 14 clusters were generated. It was found that the level of aggregation increased with greater study homogeneity when examining the keyword clusters.^[27]^ Clusters are numbered starting from #0 for the largest cluster, with the cluster number decreasing as the cluster size increases. Keyword co-occurrence and cluster analysis yielded that STP, CTS, airway stenting, difficult intubation, surgical treatment, atelectasis, trachelectomy, extracorporeal membrane oxygenation, Pierre Robin sequence, bacterial biofilm, laryngotracheal reconstruction, tracheal flow, central airway stenosis, and epithelium were the current hot topics of research in this field. A timeline view of the field’s keywords is shown in Figure 6C. It can be concluded that research hotspots such as STP, CTS, difficult intubation, surgical treatment, trachelectomy, Extracorporeal Life Support Organization – European Chapter, Pierre Robin sequence, laryngotracheal reconstruction, tracheal flow, and central airway stenosis have remained prominent and are likely to continue being significant in the future. Figure 6D displays the top 25 keywords with the strongest outbreaks, selected based on an outbreak duration of 2 years. The value of “Strength” represents the size of the burst, while “Begin” and “End” denote the start and end times of the burst, respectively. The burst duration is shown by the red line, while the time interval is indicated by the blue line. We may investigate future development tendencies and research hotspots in this sector by examining the burst words. This is particularly valuable when analyzing keywords with burst durations extending to the present, as it provides important reference and guidance. The most intense mutation is “tracheoplasty” (6.63), followed by “infants” (6.42), and the third 1 is “outcm” (6.12). The emergent words “outcm,” “extracorporeal membrane oxygenation” and “tracheal” have all persisted to the present day, and will probably continue to be a popular area of study.

**Table 5:** Top 20 high-frequency keywords and centrality in the field of surgical treatment of CTS.

Keyword	Frequency	Keyword	Centrality
Congenital tracheal stenosis	171	Infants	0.21
Slide tracheoplasty	124	Reconstruction	0.15
Management	117	Tracheoplasty	0.14
Children	107	Stenosis	0.12
Tracheal stenosis	78	Anomaly	0.11
Infants	52	Management	0.1
Reconstruction	44	Repair	0.1
Repair	32	Diagnosis	0.1
Tracheoplasty	31	Experience	0.09
Outcm	28	Congenital tracheal stenosis	0.08
Stenosis	23	Children	0.08
Experience	22	Airway stents	0.08
Resection	22	Obstruction	0.08
Complete tracheal rings	17	Tracheal stenosis	0.07
Anomaly	14	Tracheoesophageal fistula	0.07
Tracheomalacia	14	Congenital anomalies	0.06
Surgery	14	Transplantation	0.06
Airway stents	13	Resection	0.05
Pericardial patch	13	Tracheomalacia	0.05
Follow-up	12	Growth	0.05

CTS = congenital tracheal stenosis.

## 5. Key literature visual analysis

**Figure 7.**
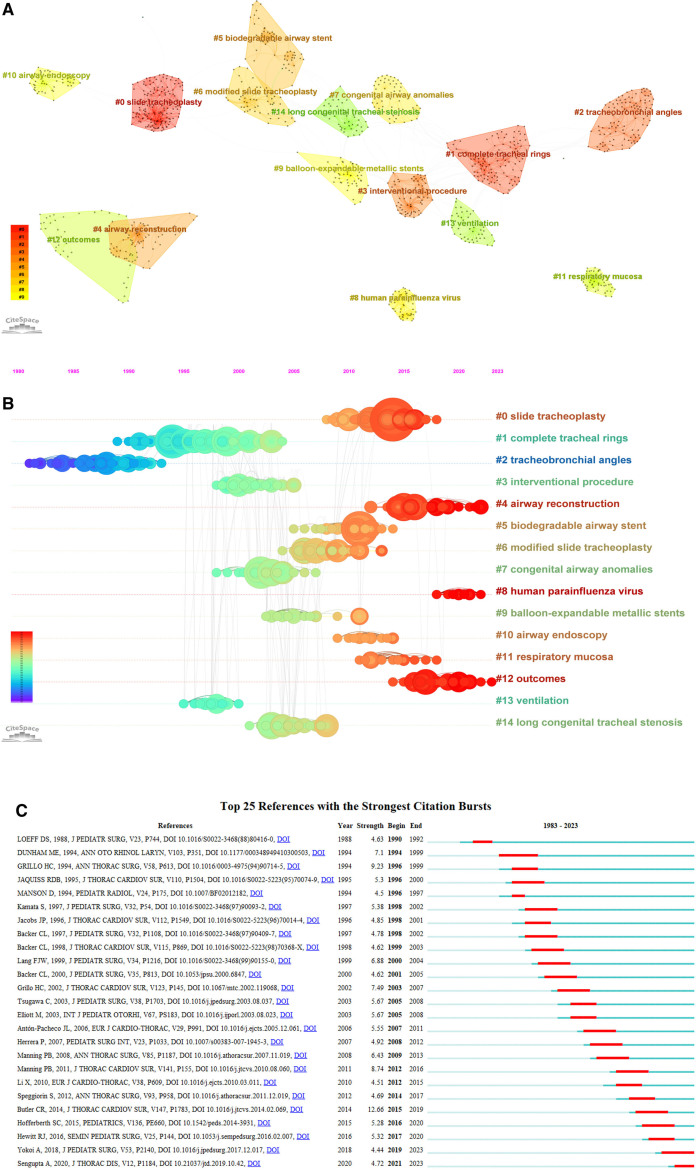
(A) Clustering of co-cited literature in the field of surgical treatment of CTS. (B) Timeline view of key literature in the field of surgical treatment of CTS. (C) Key literature emergence map in the field of surgical treatment of CTS. CTS = congenital tracheal stenosis.

Table 6 presents the 10 most frequently cited pieces of literature. The topics of study interest can be identified via reading and evaluating the literature that receives plenty of citations. It is possible to sort out the evolutionary trajectory and research hotspots in the field at the same time. Three categories may be identified based on the classification of research methods: case reports, research reviews, and cellular experiments. Items 1, 2, 3, 5, 6, 7, 8, and 10 of the cited frequency are retrospective case reports, item 9 of the cited frequency is research reviews, and item 4 of the cited frequency is in vitro cellular studies. The majority of test markers for evaluating treatment results were survival rate and long-term prognosis. Cluster analysis of co-cited literature can reveal important research hotspots in various subfields.^[33]^ Using the traditional log-likelihood ratio technique, 15 clusters were created, as illustrated in Figure 7A. The research hotspots in the field are STP, intact tracheal ring, tracheobronchial angle, interventional procedures, airway reconstruction, degradable airway stent, modified STP, congenital airway anomalies, human parainfluenza virus, balloon-expandable metal stent, airway endoscopy, respiratory mucosa, outcomes, ventilation, and congenital longitudinal tracheal stenosis. Figure 7B provides a timeline view highlighting key publications in the field, which shows that the research trends in this area are airway reconstruction, human parainfluenza virus, and outcomes, as indicated by the brighter color of the clusters. In addition, as seen in Figure 7C, the emergent citations highlight important works that are often mentioned within a specific time frame, highlighting hotspots and trends. Among the 25 emergent citations, the 1 with the highest mutation intensity was published by Butler et al in 2014, which reviewed the long-term outcomes of a total of 101 children with long-segment CTS who underwent STP in a single center over a 17-year period in terms of demographics, preoperative conditions, need for endoscopic airway intervention, procedural details, and outcome measures, and found that patient survival was 88.2% as well as that preoperative bronchial tenderness was an important factor in death and a significant risk factor for postoperative stent placement (12.66).^[30]^ Secondly, Grillo reported in his 1994 study that 4 patients with long-segment CTS treated with modified STP had their stenotic segments shortened by half, their circumference doubled, and their lumen cross-section quadrupled postoperatively, with good healing and minimal complications (9.23)^[28]^; thirdly, Manning et al in 2011 reviewed the long-term outcomes of a total of 80 patients with CTS undergoing STP over a 9-year period. It was found that longer cardiopulmonary bypass, preoperative ventilatory support, the need for significant airway re-intervention, and previous airway surgery were revealed to be predictive factors of an extended hospital stay (8.74).^[31]^

**Table 6:** Top 10 cited literature frequency rankings in the field of surgical treatment of CTS.

Author, year	Frequency	Title	Journal (IF)	JCR
Elliott MJ, 2012^[21]^	343	Stem-cell-based, tissue engineered tracheal replacement in a child: a 2-year follow-up study	Lancet (168.9)	Q1
Grillo HC, 1994^[28]^	155	Slide tracheoplasty for long-segment congenital tracheal stenosis	Annals of Thoracic Surgery (4.6)	Q1
Grillo HC, 2002^[29]^	140	Management of congenital tracheal stenosis by means of slide tracheoplasty or resection and reconstruction, with long-term follow-up of growth after slide tracheoplasty	Journal of Thoracic and Cardiovascular Surgery (6)	Q1
Butler CR, 2016^[26]^	129	Rapid Expansion of Human Epithelial Stem Cells Suitable for Airway Tissue Engineering	American Journal of Respiratory and Critical Care Medicine (24.7)	Q1
Filler RM, 1995^[19]^	121	The use of expandable metallic airway stents for tracheobronchial obstruction in children	Journal of Pediatric Surgery (2.4)	Q2
Butler CR, 2014^[30]^	111	Outcomes of slide tracheoplasty in 101 children: a 17-year single-center experience	Journal of Thoracic and Cardiovascular Surgery (6)	Q1
Manning PB, 2011^[31]^	108	One slide fits all: the versatility of slide tracheoplasty with cardiopulmonary bypass support for airway reconstruction in children	Journal of Thoracic and Cardiovascular Surgery (6)	Q1
Loeff DS, 1988^[2]^	92	Congenital tracheal stenosis: a review of 22 patients from 1965 to 1987	Journal of Pediatric Surgery (2.4)	Q2
Herrera P, 2007^[16]^	86	The current state of congenital tracheal stenosis	Pediatric Surgery International (1.8)	Q3
Backer CL, 2001^[32]^	83	Tracheal surgery in children: an 18-year review of 4 techniques	European Journal of Cardio-Thoracic Surgery (3.4)	Q1

CTS = congenital tracheal stenosis, IF = impact factor, JCR = Journal Citation Reports.

## 6. Discussion

Utilizing a bibliometric methodology, this research conducts a thorough and methodical analysis of pertinent literature published in the last 40 years about CTS surgical therapy research. By presenting the knowledge map, the study intuitively outlines the field’s knowledge structure and growth trajectory from various perspectives. The results of the study show that this field has received widespread attention. Being one of the leading nations for research, the average year of publication of scholarly publications in countries such as the US, Japan, and the UK is relatively early. They have made exceptional contributions to this field’s early study, providing a strong foundation of knowledge for further studies. In contrast, China, as the third producer country, published relevant research results only after 2000, which is in the early stage of development. China has, however, put itself on a fast track for development in this area in recent years, with a steady increase in publications that will see it overtake the US and Japan in terms of annual publications in 2020 and vault to the third rank in the world in terms of both total and annual publications. However, when it comes to publishing outputs, the ACPP of these research outputs came in second to last out of the top 10 nations, suggesting that overall research quality in Chinese publications is poor and hasn’t succeeded in gaining widespread traction in the field. It is evident that the pursuit of scholarly influence and prestige in a particular study subject requires attention to both the amount and quality of research output. In terms of publication production, Germany is only ranked seventh, but it has the highest ACPP, a sign of sophisticated research with broad recognition, frequent citations by scholars, and substantial academic impact. The impact of scanning parameters on 3D virtual endoscopy and clinical assessment of the larynx and trachea was evaluated in one of the highly cited papers. It was found that the optimal correlation between anatomical findings, diagnostic quality of axial slices, and effective longitudinal coverage in virtual endoscopy was achieved with a collimation of 3 mm and a pitch of 1.5 mm. A high-value method that allows for a nondestructive 3D display of upper airway anatomy.^[29]^ Notably, among the 10 nations with the highest number of publications, 70% are in North America and Europe, 20% in East Asia, and 10% in Australia, indicating that these regions are major centers of research in this field.

In the UK, University of London, Great Ormond Street Hospital for Children NHS Foundation Trust, and University College London have emerged as the top organizations in the subject, having not only the most publications but also the highest rankings for TC, ACPP, and H-Index. These articles, which were often published somewhat early, have significantly advanced the field’s early research. Among their widely cited works was the first case of a child undergoing stem cell tracheal replacement for long-segment CTS, which found that the grafts had local biomechanical strength, normal chest computed tomography (CT) scans, and ventilation-perfusion scans only at 18 months postoperatively and that the patient’s airway returned to normal and he returned to school at 2 years’ follow-up, underscoring the potential of the technique and the need for its further development.^[21]^ At the same time, these 3 institutions have the highest TLS, which suggests a high level of collaboration with other institutions. The University of Toronto and the Hospital for Sick Children in Canada had the eighth and ninth highest number of publications, respectively, but their ACPP were both ranked second, indicating that their high-quality scholarship is widely recognized and cited by scholars. One of the more highly cited studies was a coauthored publication by these 2 institutions on the use of an expandable metal angioplasty stent (Palmaz stent) to improve symptoms in 7 patients with congenital or other post-surgical refractory tracheal stenosis. Preliminary experience suggests its positive effect on selected lower airway blockages.^[19]^ It is noteworthy as well that the publications from Shanghai Jiao Tong University in China have a relatively late average publication year and are mainly focused on recent research areas, such as an animal trial study exploring the feasibility of a novel biodegradable magnesium alloy tracheal stent applied to CTS.^[20]^ A study to investigate the classification of CTS patients according to tracheobronchial morphology and to identify anatomical features associated with tracheobronchial anomalies and concomitant cardiovascular defects.^[32]^

Among the top 10 authors in terms of publications, Japanese, American, Chinese, and UK authors accounted for 40%, 20%, 20%, and 10% respectively. The most prolific author on the subject is Elliott of the Great Ormond Street Hospital for Children NHS Foundation Trust in the UK, who has also demonstrated his considerable academic influence and important contribution to the field by ranking first in the TC, ACPP, TLS, and H-Index. Among the earliest scholars to start studying the subject, his well-referenced paper was released on the first case of a child with long-segment CTS who underwent stem cell tracheal replacement, and at a 2-year follow-up, the patient’s airway was normalized and he returned to school.^[21]^ Studies in recent years have focused on describing the long-term outcomes of children with long-segment CTS undergoing STP in terms of demographics, preoperative conditions, need for endoscopic airway intervention, surgical details, and outcome measures,^[30]^ as well as risk factor prediction studies affecting mortality, postoperative stenting, and dysphagia.^[34]^ In addition, Backer CL and Holinger LD from Northwestern University Medical School, US, ranked lower in terms of publications but both ranked third in terms of ACPP, suggesting a high caliber of research and expertise that is well-known and frequently referenced by academics. Their collaborative, widely referenced work reviewed the short- and long-term results of 50 CTS patients treated at 1 facility, contrasting 4 distinct surgical approaches employed between 1982 and 2000. According to the study’s findings, autograft procedures were utilized for long-segment stenosis, and end-to-end anastomosis was utilized for short-segment stenosis (up to 8 rings).^[35]^ It is noteworthy that Chinese scholars accounted for 20% of the top 10 publications, but ranked first and third last in ACPP, indicating that the level of research needs to be further improved. Among them, Xu ZW from Shanghai Jiao Tong University in China is the most representative, with close communication with Zhu LM and Wang SM, and his highly cited paper is the finding that citrate-functionalized chitosan hydrogel enhances cartilage differentiation from human mesenchymal stem cells, which is a new promising tissue-engineered scaffold for regeneration of tracheal cartilage.^[36]^

When it comes to publication count, TC, TLS, and H-index, the Journal of Pediatric Surgery leads the field. One of the more frequently mentioned studies examined the cases of 22 CTS patients between 1965 and 1987 and discussed the ongoing issues with CTS therapy, including the failure to adequately evaluate the tracheobronchial tree, the failure to realize that tracheobronchography can lead to further worsening of respiratory failure, and the failure to select the appropriate surgical approach and sequence based on the evaluation of the outcome of CTS in combination with other cardiovascular diseases.^[2]^ The Journal of Thoracic and Cardiovascular Surgery is the top-ranked journal by ACPP and holds the highest impact factor among the Q1 journals in the top 10 for publication volume. A review of 11 corrective procedures performed on patients with various forms of tracheal stenosis and a retrospective assessment of the hospital course, complications, and long-term results were among its well-known publications, which found that STP had good short- and long-term results. For short-segment congenital stenosis can be effectively treated by resection and anastomosis.^[37]^ The most cited Q1 journal with the greatest impact factor is the European Journal of Cardio-Thoracic Surgery, whereas the Q1 journal with the highest H-index is the Annals of Thoracic Surgery. This suggests that the publications published in these 2 prestigious and authoritative journals have a high academic reference value. You should submit your work to these highly prolific journals first when publishing research on this topic, and you should search for relevant literature in these highly referenced journals’ proceedings.

High-cited literature often indicates excellent research, has a significant scholarly impact, and sheds light on the main areas of interest for that field’s research.^[38]^ As a result, examining the highly referenced literature can offer some preliminary understanding of the field’s research trends and directions. In terms of morbidity and preoperative predictive risk factors, Butler et al (2014) reviewed the long-term outcomes of a total of 101 children with long-segment CTS who underwent STP in a single center from 1995 to 2012. The results found that the overall patient survival rate was 88.2% and that preoperative bronchial tenderness was a significant risk factor for death and postoperative stent placement.^[30]^ Manning et al (2011) reviewed the long-term results of 80 CTS patients who underwent STP between 2001 and 2009 and discovered a 5% death rate. Preoperative ventilatory support, prior airway surgery, longer cardiopulmonary bypass, and the requirement for significant airway re-intervention were found to be predictive of a longer hospital stay.^[31]^ A similar report was made by Loeff et al (1988) who reviewed the overall mortality rate of 77% in 22 patients with CTS who underwent surgery from 1965 to 1987.^[2]^ It can be found that with the popularization of new surgical modalities and the improvement of related theories, the mortality rate of surgical treatment of CTS patients has gradually decreased. In terms of surgical treatment modalities, Elliott et al (2012) published the first pediatric case of long-segment CTS with lung sling undergoing stem cell tracheal replacement. It was found that evidence of significant cytologic recovery of the epithelium did not occur until 1 year. The grafts did not have local biomechanical strength and normal ventilation-perfusion until 18 months postoperatively, and the airway returned to normal after 2 years.^[21]^ Epithelial cells are usually obtained by culturing for multiple generations in serum-free bronchial epithelial growth medium^[39]^ which is an inefficient output for regenerative applications and poor ciliated epithelial function.^[40,41]^ Clinical experience, however, has demonstrated that it is crucial to restore mucociliary clearance from the lungs following tracheal transplantation. This is a result of the distal anastomosis holding onto secretions, which can lead to infection and airway obstruction. This places a requirement on the inoculation of high-density epithelial cells.^[42]^ Butler et al (2016) demonstrated for the first time that a significant number of functional airway basal epithelial cells with the efficiency needed for clinical transplantation and appropriate for tracheal restoration may be produced by using 3T3-J2 feeder cells in conjunction with Rho-associated coiled-coil kinase inhibition.^[26]^ Filler et al (1995) reported the implantation of an expandable metallic angioplasty stent (Palmaz stent) into 7 patients with symptomatic refractory tracheal stenosis after congenital or other surgery. Preliminary experience suggests that the stent is characterized by immutable placement and easy removal and has a positive effect on selected lower airway blockages.^[19]^ Grillo (1994) reported that 4 patients with long-segment CTS treated with a modified STP had the stenotic segments halved in length, the circumference doubled, and the lumen cross-section quadrupled postoperatively, with good healing and very few complications.^[28]^ This is the first time since Tsang 1989 study. This is the first report of the successful use of this method since it was first described by Tsang team in 1989.^[43]^ Grillo modified the initial surgical approach by incising the upper section of the trachea backward and the lower section forward. Grillo et al (2002) corrected stenosis in 11 patients with different types of CTS. It was found that STP had good short and long-term results, and short-segment congenital stenosis could be effectively treated with resection and anastomosis.^[37]^ Backer et al (2001) reviewed the short- and long-term outcomes of 50 patients with CTS, and the study concluded that end-to-end anastomosis was used for short-segment stenoses (up to 8 loops), whereas the autograft technique was used for long-segment stenoses.^[35]^ On this basis, a literature review study by Herrera et al (2007) concluded that there is no one standard technique for the repair of CTS, it should be customized for each patient and respiratory lesion. Anastomosis and segmental resection work best for short-segment stenosis, whereas STP works best for long-segment stenosis.^[16]^ STP is reconstructed using natural gas tube tissue that has normal epithelium for immediate stabilization. Much of the lateral blood supply to the trachea is preserved, reducing the probability of loss of viability due to inadequate blood supply. The procedure is also simpler to perform because cardiopulmonary bypass is usually not required.

Cluster analysis of keyword co-occurrence showed that the main research areas were the clinical efficacy of selecting individualized surgical procedures (costal cartilage patch graft, pericardial patch graft, tracheal patch autograft, stenotomy end-to-end anastomosis, and sliding tracheoplasty) for the treatment of CTS in combination with other cardiovascular diseases, and the use of extracorporeal circulatory equipment (extracorporeal membrane pulmonary oxygenation) as an adjunct in order to reduce the mortality rate of the surgery. Cluster analysis of the key literature indicated that the main research areas of interest were the clinical efficacy of selecting individualized surgical sequences for the treatment of CTS combined with other cardiovascular diseases and the exploration of preoperative predictive risk factors by reviewing patient cases. In the future, the reliability and feasibility of novel metrics to objectively assess tracheal flow function before and after surgery, new bioabsorbable scaffolds with longer degradation times and increased radial expansion, the exploratory application of new technologies such as visual surgery and artificial intelligence, to decrease the mortality rate and the need for reoperation, which can provide useful support for treating the efficacy of CTS.

In recent years, the treatment of CTS has made great progress. However, there is still a lack of unified standards regarding the typing of CTS. So far, Cantrell typing is still used^[44]^ and Anton-Pacheco staging is the most widely used. Table 7 statistically shows the basis and description of the main CTS subtypes.^[45–50]^ The common surgical treatments currently available are, rib cartilage,^[9,10]^ pericardium,^[11,12]^ trachea,^[13]^ esophagus,^[14]^ periosteum^[15]^ patch grafting, resection of a stenotic segment of the trachea with end-to-end anastomosis,^[16]^ sliding tracheoplasty,^[17]^ anterior tracheal wall suspension plasty,^[18]^ expandable metal airway stenting,^[19]^ biodegradable material airway stent placement^[20]^ and stem cell tracheal replacement.^[21]^ The mainstay technique in the treatment of short-segment CTS is tracheal stenosis resection with end-to-end anastomosis, while slide anastomosis is the mainstay technique in the treatment of long-segment CTS.^[17,22]^ Table 8 describes the characteristics of the main types of surgical treatment for CTS. However, surgical treatment of CTS is often complicated by the presence of congenital heart defects, such as atrial septal defects, ventricular septal defects, tetralogy of Fallot, arterial catheterization, and vascular rings. Common vascular loops include pulmonary artery slings, double aortic arches, and right arch vagus left subclavian artery. The most common of these are combined with a pulmonary artery sling to form a “ring sling complex.”^[8]^ The current preferred approach is to integrate the repair of both flaws when they are present, as this is a practical solution. Questions have been raised about the optimal timing and sequencing of the procedures, with some suggesting staging the repair for individuals who have complicated concomitant cardiac diseases.^[17,51]^ For example, Okamoto T et al conducted a retrospective comparison of the surgical results of 32 CTS patients, 27 who underwent concomitant repair of cardiac anomalies, and 5 who underwent staged repair. The authors found that CTS involving complex cardiac lesions or requiring prolonged extracorporeal circulatory support could benefit from staged repairs, whereas simpler associated cardiac defects should be repaired at the same time as tracheal reconstruction.^[52]^ Regardless of the type of related cardiac abnormalities, data from the previous 20 years indicates that patients with co-morbidities can have both lesions corrected simultaneously with good short- and medium-term results.^[53]^ A retrospective assessment of 21 patients who had their cardiac abnormalities and tracheal stenosis repaired simultaneously was carried out by Mainwaring et al The findings indicate that no patient needed a significant re-intervention of the bronchi or trachea, indicating that complicated cardiac anomalies may and need to be fixed during tracheal surgery.^[54]^ Xue et al examined the outcomes of 43 juvenile patients – 31 pulmonary artery slings, 5 tetralogy of Fallot cases, 4 cases of ventricular septal defect, 4 cases of atrial septal defect, 2 cases of double aortic arch, and 1 case of pulmonary artery atresia with ventricular septal defect – who underwent combined tracheal and cardiac surgery over a 13-year period. Five cases underwent simple trachelectomy, 8 cases underwent autologous tracheal transplantation and 30 cases underwent sliding tracheoplasty. It was discovered that the complication rate was not raised by concurrently correcting the related congenital cardiac abnormalities.^[55]^ Furthermore, the study team at Great Ormond Street Hospital for infants reported on 72 infants who had long-segment CTS (combined cardiac and tracheal repair) between 1995 and 2012, and they established an overall survival rate of 88.2%.^[30]^ In conclusion, available data indicates that the best results are obtained when all abnormalities are repaired simultaneously by employing a median sternotomy, extracorporeal circulation, and versatile sliding tracheoplasty for tracheal reconstruction. Staged repair may be considered only when mild tracheal stenosis is present.

**Table 7:** Description and basis of major CTS subtypes.

Typing category	Basis of typing	Description of typing
Cantrell typing^[44]^	Anatomical malformations of the trachea	Diffuse, trachea to carina showing uniform stenosis
		Funnel-shaped, with the trachea thick at the upper end and tapering downward to the carina or above the carina, like a funnel
		Segmental, restrictive stenosis with hourglass narrowing of a small segment (1 to 5 cm) of the trachea
Hoffer typing^[45]^	narrowing length	Limited stenosis: tracheal stenosis < 50% of length
		Long-segment stenosis: 50% to 80% of the length of the tracheal stenosis
		Diffuse stenosis: >80% of tracheal stenosis length
Anton-Pacheco typing^[46]^	clinical manifestation	Mild: occasional or no clinical manifestations
		Medium: clinical signs but no respiratory distress
		Medium: clinical signs but no respiratory distress
	combined malformation	A: Combination of other malformations
		B: No other deformities
Anand typing^[47]^	narrow caliber	Light (<70%), Medium (71%–90%), Heavy (>90%)
	narrowing	Under the vocal folds, neck, chest
	narrowing length	Light (<1 cm), medium (1% to 3 cm), heavy (>3 cm)
Myer typing^[48]^	narrow caliber	Light (<50%), medium (51%–70%), heavy (>70%)
Freitag typing^[49]^	Etiology and tracheal morphology	Type I, stenosis due to granulation or tumor in the canal lumen
		Type II, extracavitary compression
		Type III, stenosis due to tracheal twisting
		Type IV, stenosis due to scar tissue
		Type V, sheath stenosis
		Type IV, stenosis due to softening and collapse of the tracheal wall
		Type VII, sudden stenosis i.e. stenosis of normal caliber of upper and lower trachea
		Type VIII, Seat Clock Narrow
	narrowing	Stenosis of the upper, middle and lower third of the trachea and the left and right bronchi
	narrow caliber	0% to 25%, 26% to 50%, 51% to 75%, 76% to 90%, 90% to complete atresia
Speggiorin typing^[50]^	Abnormalities in the bronchial tubes	Type I, normal bronchial bifurcation type
		Type II with right upper bronchial bifurcation
		Type III, trigeminal branching at the bulge
		Type IV, unilateral bronchial tree type i.e. unilateral pulmonary
Hu typing^[35]^	Tracheobronchial morphology	Ⅰ Type A, normal tracheobronchial dendritization
		Ⅰ Type B with right upper bronchial bifurcation
		Type II, trigeminal branching at the bulge
		Type III, typical bridging bronchus
		Type IV A, atypical bridging bronchus, right main bronchus only as short bronchial diverticulum
		Type IV B, atypical bridging bronchus, and absence of bronchial diverticulum

CTS = congenital tracheal stenosis.

**Table 8:** Description and characteristics of the main types of surgical treatment of CTS.

Name	Indications	Concrete method	Clinical characteristics
Cartilage,^[10]^ Pericardium,^[12]^ Trachea,^[13]^ Esophagus,^[14]^ Periosteum^[15]^ patch grafting	Long segmental stenosis for repair of the anterior and lateral walls of the trachea.	A variety of tissue materials including cartilage, pericardium, trachea, esophagus, and periosteum are used to repair the anterior and lateral walls of the trachea by expanding the inner diameter of the trachea with a patch to heal the stenosis.	The mainstay of early surgical treatment of long-segment CTS. The short-term postoperative outcome is good, but there is a high incidence of late granulation tissue proliferation, causing restenosis.
Tracheal stenosis resection with end-to-end anastomosis^[16]^	Short-segment CTS (<30% total tracheal length or < 6 tracheal rings)	The stenotic segment is identified, the stenotic trachea is completely resected, and direct anastomosis is performed between the upper and lower severed ends. Sutures can be interrupted or continuous.	Mainstream technology for the treatment of short-segment CTS.
Sliding tracheoplasty^[17]^	This technique is preferred for all CTS, especially long-segment CTS, bronchial bridge deformities, or combined branch stenosis.	The midpoint of the stenotic segment of the trachea is transected, the anterior wall of the upper trachea and the posterior wall of the lower trachea are dissected longitudinally, and the openings at the upper and lower ends are then trimmed to a spatula shape and “slid” together. In cases of combined branch tracheal stenosis, a “side-to-side slide” anastomosis technique is used.^[17]^	Currently the dominant technique for the treatment of long-segment CTS. Reconstruction using natural gas tube tissue preserves much of the lateral blood supply, reduces postoperative granulation, avoids restenosis, and is simpler to implement as cardiopulmonary bypass is usually not required postoperatively.
Anterior tracheal wall suspension molding^[18]^	Tracheal reconstruction in which localized softening of the anterior tracheal wall is still evident	Under bronchoscopic guidance, the anterior wall of the softened segment of the trachea was preplaced with a long-lasting absorbable suture and suspended behind the sternum.	Prevents tracheal softening
Expandable Metal Airway Stent Placement Plasty^[19]^	Tracheal wall softening and collapse is severe or anterior wall suspension is ineffective.	Under fluoroscopy, a balloon-expandable stent is inserted through the bronchoscope into the airway stenosis and the balloon is then inflated.	Prevents tracheal softening and collapse with incomplete epithelialization of early and late stents with granulation tissue patches. Characterized by fixed placement and easy removal.
Airway stent placement and molding with degradable materials^[20]^	After tracheoplasty, the wall of the trachea or tracheal branches is severely softened and collapsed, or the softened segment is long and the anterior wall suspension is not effective.	Under bronchoscopic guidance, an absorbable external tracheal stent was placed outside the softened segment of the trachea, and the anterior, lateral, or posterior wall of the traction trachea was closed with absorbable sutures and knotted to secure the external stent.	Prevents tracheal softening and collapse, and the material is biodegradable, reducing the need for follow-up surgery.
Stem cell tracheal replacement^[21]^	Emergencies after failure of other stenting or surgical treatments	By decellularizing donor tracheal stents and seeding them with bone marrow mesenchymal stem cells, together with autologous epithelial patches, the trachea is replaced as a whole.	Immunosuppression is avoided, eliminating the need for repeat surgical interventions.

CTS = congenital tracheal stenosis.

With the boom in interdisciplinary research, new research opportunities have emerged in the field, many scientific techniques such as CFD, bioabsorbable scaffolds, and visual surgery have been rapidly developed in recent years and are widely used in the clinic, playing an indispensable role in the choice of treatments. The degree of tracheal stenosis is typically used to determine the severity of CTS.^[56,57]^ The severity of CTS should also be evaluated based on the degree of airflow patency, as clinical experience has demonstrated that assessments based on the degree of stenosis may not necessarily represent the actual respiratory condition. CFD is a computational 3-dimensional modeling of airway flow through individual imaging techniques.^[24]^ Objective assessment of the pathophysiology and postoperative benefits of various airway stenoses in children from a fluid dynamics perspective has attracted much attention. A study by Takeishi et al found that postoperative enlargement of the cross-sectional area by CFD assessment did not always contribute to the restoration of postoperative tracheal flow.^[58]^ Morita et al first proposed energy flux as a useful parameter for surgical evaluation of CTS. After using CFD to examine 15 children with CTS, they discovered that energy flux was positively connected with the clinical respiratory condition both before and after surgery and that it more correctly represented the clinical respiratory status than the degree of stenosis.^[25]^ In addition, Bao et al have applied CFD assessment to CTS combined with obstructive sleep apnea syndrome in children to construct objective indications for CFD-based assessment of CTS combined with various tracheal abnormalities to make better therapeutic decisions preoperatively. Bioresorbable materials are polymers that degrade in vivo under the guidance of cells.^[59]^ Common polymer implants initially undergo hydrolysis, producing byproducts that are excreted in the urine (e.g., glycolate) or are further broken down through the Kreb cycle, ultimately producing carbon dioxide and water (e.g., glycine and lactate).^[60]^ Recently, increasingly, experiments on humans and animals have begun using bioabsorbable endotracheal scaffolds, using materials such as polydioxanone,^[61–63]^ polyglycolic acid/poly-L-lactide co-polymer (VICRYL®),^[64,65]^ polycaprolactone,^[66,67]^ poly-L-lactic acid^[68,69]^ and magnesium alloys.^[20,70]^ Pediatric airway blockage that is not infectious may benefit from the use of bioresorbable scaffolds as a therapy option. It would be beneficial to investigate new materials that have longer degradation durations and stronger radial expansion pressures in order to lower the risk of stent fragment foreign body problems and lessen the requirement for follow-up surgery. While CTS procedures are typically performed using CT and endoscopic assistance, several studies of new visual surgical techniques are currently emerging in the field. Kehl et al Using a specialized open-source platform (3D Slicer), secondary processing of enhanced dual-source CT scans produced 3-dimensional (3D) representations that made the link between the trachea and the innominate artery easily understandable. In the context of congenital heart disease, this offers a great chance for the diagnosis, planning of therapy, and follow-up of patients with tracheal stenosis associated with vascular issues.^[71]^ Shaver et al proposed a new technique using ultrasound to visualize the esophageal lumen and identify the needle that punctures the posterior wall of the trachea for placement of a tracheoesophageal prosthesis.^[72]^ Overall, with the emergence of new technologies and approaches that not only provide innovative ideas for clinical therapeutic applications but also actively improve treatment options for CTS, there is hope for further reductions in CTS mortality in the future.

## 7. Study limitations

This study has several limitations, including the restriction to English-language literature from the core dataset of the WoS database. This may have resulted in the exclusion of valuable studies from other databases or in different languages. However, it is noteworthy that the WoSCC is considered by many to be one of the datasets that is most frequently utilized in bibliometric research.^[73–75]^ For the time being, there is no standardized procedure for selecting the time dividing, filtering criterion (threshold), or cropping technique for creating visual maps. The quantity of papers in the divided time zones varies depending on the time division. Due to the screening parameters, there are unequal numbers of extracted publications with the most citations in each time division after segmentation. There is a chance of bias since the path-finding algorithm’s pruning of the research network becomes inconsistent as a result of the pruning strategy. In the future, analysis incorporating more clinical perspectives will help improve the depth and accuracy of the content.

## 8. Conclusion

This study provides a novel perspective for quickly understanding the field of surgical treatment of CTS, as it is the first bibliometric and graphical examination of studies conducted in this field from a variety of perspectives throughout the previous 40 years. There is still a lot of room for growth in this subject, according to recent studies. The most influential countries, journals, institutions, and authors are the US, Journal of Pediatric Surgery, Great Ormond Street Hospital for Children NHS Foundation Trust, and Elliott. However, there are still many opportunities and challenges in the surgical treatment of CTS research, including clinical efficacy study of selecting the optimal timing and sequence of surgical procedures for the treatment of CTS combined with cardiovascular disease, the use of extracorporeal circulatory devices to assist in the reduction of surgical mortality, and the explore preoperative predictive risk by reviewing patient cases to explore preoperative predictive risk factor studies. Meanwhile, future studies should focus on exploring the reliability and feasibility of novel metrics to objectively assess tracheal flow function before and after surgery, new bioabsorbable scaffolds with longer degradation times and increased radial expansion, and exploring the use of new technologies such as visual surgery and artificial intelligence. The knowledge map that is used to present this study offers a thorough and organized overview of the vast and detailed literature resources pertaining to the topic of surgical treatment of CTS. This encourages further research efforts by making it easier for academics to comprehend the field’s possibilities and progress.

## Acknowledgments

The authors thank all the authors of the original studies included in this analysis.

## Author contributions

**Conceptualization:** Jie Xu, Meng Chen.

**Data curation:** Jie Xu, Meng Chen, Xin Wang.

**Formal analysis:** Meng Chen, Xin Wang.

**Funding acquisition:** Xiaobing Luo.

**Investigation:** Jie Xu, Xiaobing Luo.

**Methodology:** Jie Xu, Xiaobing Luo.

**Project administration:** Xin Wang.

**Resources:** Xiaobing Luo.

**Software:** Jie Xu, Meng Chen.

**Supervision:** Xiaobing Luo.

**Validation:** Xiaobing Luo.

**Visualization:** Jie Xu.

**Writing – original draft:** Jie Xu, Meng Chen.

**Writing – review & editing:** Jie Xu, Meng Chen.

## Supplementary Material


