# Supplementary material for: Global research hotspots and trends in the field of surgical treatment of congenital tracheal stenosis in infants and children over the past 40 years: A bibliometric and visualization study
Source: Medicine (Baltimore). 2025 Jul 4;104(27):e43143. doi: 10.1097/MD.0000000000043143 (PMC12237391; doi:10.1097/MD.0000000000043143)
Supplement: Supplementary file 1 [file medi-104-e43143-s001.docx]

Supplementary table 1

Table S1 Search strategy of Web of science Collection

| #1 | TS=(Tracheal Stenosis OR Tracheal Stenoses) |
| --- | --- |
| #2 | TS=(Congenital OR Connatal) |
| #3 | TS=(Infant OR Children OR Child) |
| #4 | TS=(Surgical OR Surgery OR Surgical Procedure OR Operative Procedure OR Operative Surgical) |
| #5 | #1 and #2 and #3 and #4 |
