# Supplementary material for: Global research hotspots and trends in the field of surgical treatment of congenital tracheal stenosis in infants and children over the past 40 years: A bibliometric and visualization study
Source: Medicine (Baltimore). 2025 Jul 4;104(27):e43143. doi: 10.1097/MD.0000000000043143 (PMC12237391; doi:10.1097/MD.0000000000043143)
Supplement: Supplementary file 2 [file medi-104-e43143-s002.docx]

Supplementary table 2

Table S2 Specific parameter information used in Citespace

| **Option** | **Parameter** |
| --- | --- |
| Language | English |
| Publication Year | 1983.01.01-2024.01.24 |
| Document Type | Article, Review |
| **Filters** | Default parameters |
| Show the Largest Connected Component Only | Yes |
| Show Citation/Frequency Burst | Yes |
| **Text Processing** | Default parameters |
| **Term source** | Default parameters |
| Title | Yes |
| Abstract | Yes |
| Author Keywords (DE) | Yes |
| Keywords Plus (ID) | Yes |
| **Term Type** | Default parameters |
| Noun Phrases | No |
| Burst Terms | No |
| **Node Types** |  |
| Author/Institution/Country/Keyword/Category/Reference/Cited Author/Cited Journal | Options |
| **Links** | Default parameters |
| Strength | Cosine |
| Scope | Within Slices |
| Selection Criteria | Top N (50) |
| **Pruning** |  |
| Pathfinder | Yes |
| Pruning sliced networks | Yes |
| Minimum Spanning Tree | No |
| Pruning the merged network | No |
| **Visualization** | Default parameters |
| Cluster View - Static | Yes |
| Show Networks by Time Slices | No |
| Cluster View - Animated | No |
| Show Merged Network | Yes |
| **Clustering** | Default parameters |
| clustering and labeling | All in One |
| **Burstness** |  |
| Configure the detection model |  |
| f(x)=αe^-αx^,α_1_/α_0_ | 2.0 |
| α_i_/α_i-1_ | 2.0 |
| The Number of States | 2 |
| γ[0,1] | 1.0 |
| Minimum Duration | 5 |
| Burst items found | 30 |
