# Supplementary material for: Global research hotspots and trends in the field of surgical treatment of congenital tracheal stenosis in infants and children over the past 40 years: A bibliometric and visualization study
Source: Medicine (Baltimore). 2025 Jul 4;104(27):e43143. doi: 10.1097/MD.0000000000043143 (PMC12237391; doi:10.1097/MD.0000000000043143)
Supplement: Supplementary file 3 [file medi-104-e43143-s003.docx]

Supplementary table 3

Table S3 Specific parameter information used in VOSviewer

| **Option** | **Parameter** |
| --- | --- |
| Language | English |
| Publication Year | 1983.01.01-2024.01.24 |
| Document Type | Article, Review |
| Minimum number of occurrences of a keyword | 5 |
| Normalization Method | Association strength |
| **Layout** | Default parameters |
| attraction | 2 |
| repulsion | 0 |
| Random strats | 1 |
| Max.iterations | 1000 |
| Initial step size | 1.00 |
| Step size reduction | 0.75 |
| Step size convergence | 0.001 |
| Random seed | 0 |
| **Clustering** | Default parameters |
| resolution | 1.00 |
| min.cluster size | 1 |
| Merge small clusters | Yes |
| Random starts | 10 |
| Iterations | 10 |
| Random seed | 0 |
| **Visualization** |  |
| Scale | 1.00 |
| Weights | Occurrences |
| Scores | Avg. pub.year |
| **Labels** |  |
| Size variation | 0.50 |
| Max. length | 30 |
| Font | Open Sans |
| **Lines** |  |
| Size variation | 0.50 |
| Min. strength | 0 |
| Max. lines | 1000 |
